# Wearable-Based Mobile Health App in Gastric Cancer Patients for Postoperative Physical Activity Monitoring: Focus Group Study

**DOI:** 10.2196/11989

**Published:** 2019-04-23

**Authors:** Jin-Ming Wu, Te-Wei Ho, Yao-Ting Chang, ChungChieh Hsu, Chia Jui Tsai, Feipei Lai, Ming-Tsan Lin

**Affiliations:** 1 Department of Surgery National Taiwan University Hospital National Taiwan University Taipei Taiwan; 2 Department of Surgery, College of Medicine, National Taiwan University Taipei Taiwan; 3 Graduate Institute of Biomedical Electronics and Bioinformatics National Taiwan University Taipei Taiwan

**Keywords:** telemedicine, exercise, perioperative care, gastrectomy, stomach neoplasms

## Abstract

**Background:**

Surgical cancer patients often have deteriorated physical activity (PA), which in turn, contributes to poor outcomes and early recurrence of cancer. Mobile health (mHealth) platforms are progressively used for monitoring clinical conditions in medical subjects. Despite prevalent enthusiasm for the use of mHealth, limited studies have applied these platforms to surgical patients who are in much need of care because of acutely significant loss of physical function during the postoperative period.

**Objective:**

The aim of our study was to determine the feasibility and clinical value of using 1 wearable device connected with the mHealth platform to record PA among patients with gastric cancer (GC) who had undergone gastrectomy.

**Methods:**

We enrolled surgical GC patients during their inpatient stay and trained them to use the app and wearable device, enabling them to automatically monitor their walking steps. The patients continued to transmit data until postoperative day 28. The primary aim of this study was to validate the feasibility of this system, which was defined as the proportion of participants using each element of the system (wearing the device and uploading step counts) for at least 70% of the 28-day study. “Definitely feasible,” “possibly feasible,” and “not feasible” were defined as ≥70%, 50%-69%, and <50% of participants meeting the criteria, respectively. Moreover, the secondary aim was to evaluate the clinical value of measuring walking steps by examining whether they were associated with early discharge (length of hospital stay <9 days).

**Results:**

We enrolled 43 GC inpatients for the analysis. The weekly submission rate at the first, second, third, and fourth week was 100%, 93%, 91%, and 86%, respectively. The overall daily submission rate was 95.5% (1150 days, with 43 subjects submitting data for 28 days). These data showed that this system met the definition of “definitely feasible.” Of the 54 missed transmission days, 6 occurred in week 2, 12 occurred in week 3, and 36 occurred in week 4. The primary reason for not sending data was that patients or caregivers forgot to charge the wearable devices (>90%). Furthermore, we used a multivariable-adjusted model to predict early discharge, which demonstrated that every 1000-step increment of walking on postoperative day 5 was associated with early discharge (odds ratio 2.72, 95% CI 1.17-6.32; *P*=.02).

**Conclusions:**

Incorporating the use of mobile phone apps with wearable devices to record PA in patients of postoperative GC was feasible in patients undergoing gastrectomy in this study. With the support of the *mHealth* platform, this app offers seamless tracing of patients’ recovery with a little extra burden and turns subjective PA into an objective, measurable parameter.

## Introduction

### Background

Physical activity (PA) is a well-established parameter to not only assess the general condition but also monitor the recovery of patients [1,2]. Regular PA can be protective against the risk of gastric and breast cancers [3,4]. Although the double-labeled water method is considered to be the gold standard for assessing total energy expenditure (to reflect PA), it is rarely used because it is expensive, equipment dependent, and time consuming [5,6]. Therefore, other modalities, including self-report questionnaires, self-report activity diaries, direct observation, and devices (accelerometers, pedometers, or armbands), have been implemented to measure PA [6].

Gastric cancer (GC) is the fourth leading cancer worldwide and the second leading cause of cancer-related mortality [7]. In Taiwan, nearly 3800 new cases of GC are reported each year in patients with a median age of 70 years. Complete surgical resection and endoscopic submucosal resection are the only curative therapies that provide better long-term survival. Nevertheless, gastrectomy-related stress and discomfort adversely affect PA and quality of life immediately postoperatively and last for up to 6 months [8], contributing to poor outcomes or early recurrence, particularly in cases with advanced-stage GC with obstruction of the gastrointestinal tract and malnutrition [9,10]. Furthermore, PA declines more markedly in patients who have undergone gastrectomy and are receiving postoperative chemotherapy or chemoradiation [11]. Moreover, patients with GC encounter significant functional impairments and decreased quality of life because of decreased PA and increased gastrointestinal symptoms [12]. Despite the deterioration in physical function, regular PA (to strengthen muscle power, which leads to improved physical function), proper nutritional intervention (to improve food intake, which results in weight gain), and mental support (to preserve self-esteem and maintain social activity) may help in restoring the patients’ health status and improving quality of life [13].

Traditional perioperative care depends on medical professionals asking patients about the progression of their PA; however, these self-report measures are not only unreliable in aged adults with cognitive impairment [14] but also time consuming in processing the data [15]. For the purpose of clinical research, self-report questionnaires are the most common method for PA assessment, and they have the advantage of cost-effectiveness and ease of administration [6]. However, compared with using devices for recording PA, potential disadvantages of self-report questionnaires are that they may be less reliable in measuring light or moderate PA and may also be affected by external factors, such as social desirability, age, complexity of the questionnaire, and the participants’ recall ability [16,17]. Furthermore, 4 key categories of aging barriers are associated with the use of mobile health (mHealth) in aged adults, including barriers in cognition, motivation, physical ability, and perception [18]. As GC typically comprises an aged population (median age >65 years) with the potential for developing cognitive or memory impairments, it is important to select an easy-to-follow PA device to use in clinical research for this population.

mHealth is a scalable and flexible platform that can assist the practice of medicine and public health with the support of mobile devices [19-21]. Several studies have demonstrated that mHealth technology has improved clinical outcomes in medical patients by improving the control of cardiac function and glycemic hemostasis, enhancing medication compliance, and shortening hospital stay [22-25]. Although the experience of using the mHealth app in surgical care is limited [26,27], it is suggested that surgical patients can benefit from this new technology support and restore the critical decline in physical and medical functions. The mHealth system and its associated mobile apps support many theory-based techniques that have shown to increase PA in behavioral interventions [28-31], with self-monitoring being the most important element associated with success of the intervention [29]. With the support of technology-based trackers, patients are encouraged to self-monitor, and wearing automatic recorders of PA reduces the burden. Furthermore, 1 study investigated the accuracy in step counting among commercially available wearable devices, showing that most devices did not overcount or undercount steps [32]. These findings are particularly important for clinical interventions using such wearable devices for clinical research.

### Objectives

Five years ago, our team worked with bioinformatics developers at our university to create a new first-generation mobile phone/tablet app (SurgeryDiary) to accelerate recovery in patients who have undergone gastrointestinal surgery [33]. This next-generation app implemented a wearable device to track daily PA of patients with GC. This pilot study focused on the feasibility and clinical value of the second-generation app in patients with GC. In addition, this study determined the correlation between PA variables collected from the wearable device and outcomes and illustrated how the device can be used to estimate patient recovery.

## Methods

### Study Population

In this study, eligible participants were adult inpatients (aged ≥20 years) on the general surgery service of an academic teaching hospital. We enrolled patients who were undergoing laparoscopic or open gastrectomy for GC at our institution from January 2016 to December 2017. All patients fulfilling the inclusion criteria were approached to participate in this study. Notably, patients were excluded if they had preoperative walking disorders (paralysis or hemiplegia) or prolonged stay in the intensive care unit (ICU). During the study period, 50 patients were screened; of these patients, we enrolled 43 in this study, excluding 7 with a prolonged ICU stay. This study protocol was approved by the institutional review board of the National Taiwan University Hospital (201412040RIND). A research assistant helped the enrolled patients to install the app on their smartphones and instructed them on how to use the app preoperatively. Before the enrollment of this study, the research assistant would evaluate the patients’ familiarity with wearable devices and smartphones. If the patients were not confident about using these devices, we would provide further instruction to their caregivers.

Data of patients’ demographics and oncological factors were obtained by 2 medical professionals after reviewing charts (discharge summaries, imaging reports, and pathological reports). Regarding comorbidities, we collected the following data on the comorbidity of patients before gastrectomy using the International Classification of Diseases, 9th Revision, Clinical Modification (ICD-9-CM) codes: anemia (ICD-9-CM: 285.x), myocardial infarction (410.x and 412.x), mild liver disease (571.2 and 571.4-571.6), hyperlipidemia (272.0-272.2), diabetes mellitus (250.0-250.3 and 250.7), chronic obstructive pulmonary disease (490.x-496.x), renal failure (584.x-586.x), and hypertension (401.x-405.x). Next, we used the Charlson comorbidity index to calculate baseline comorbidity scores for each patient [34]. This was used to compare the baseline comorbidity between the 2 groups. Moreover, we used the Eastern Cooperative Oncology Group performance status to represent the baseline daily living ability [35]. Higher scores in both scales implied that the patients had poor medical and physical functions. Furthermore, the American Society of Anesthesiologists (ASA) score, which ranges from 1 to 5, assesses the preoperative physical status of patients [36]. The definition of ASA 1, 2, 3, 4, and 5 is “a normal healthy patient,” “a patient with mild systemic disease,” “a patient with severe systemic disease,” “a patient with severe systemic disease that is a constant threat to life,” and “a moribund patient who is not expected to survive without the operation,” respectively. For the oncological variables, including cancer histology and cancer stage, the American Joint Committee on Cancer staging was implemented to determine cancer stages of this study’s population [37]. Satisfaction survey of the wearable device and app was measured using items adapted from a previous study [38], including 5 questions (patient comfort using the wearable device, if the patient would continue to wear the device, if it was convenient for the patient to use the app, if the app was user friendly, and if the patient would recommend this app). All responses were made on a Likert-type scale from 1 (strongly disagree) to 5 (strongly agree) as the study period was completed.

### The App

**Figure 1:**
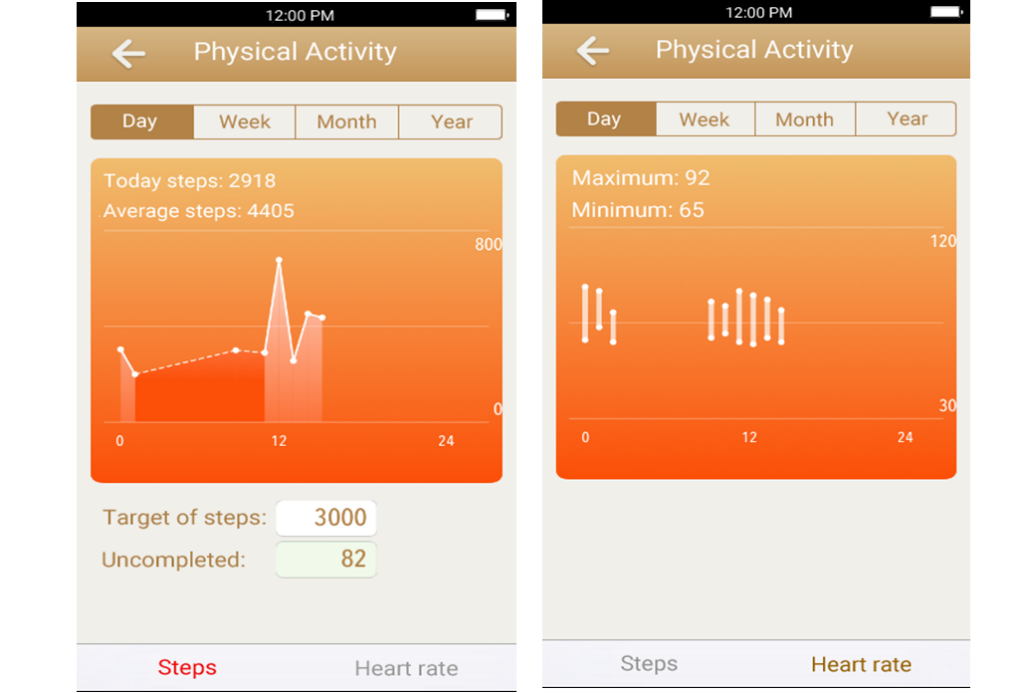
Screenshots of the app showing the number of walking steps (left) and heart rates (right) at different periods.

**Figure 2:**
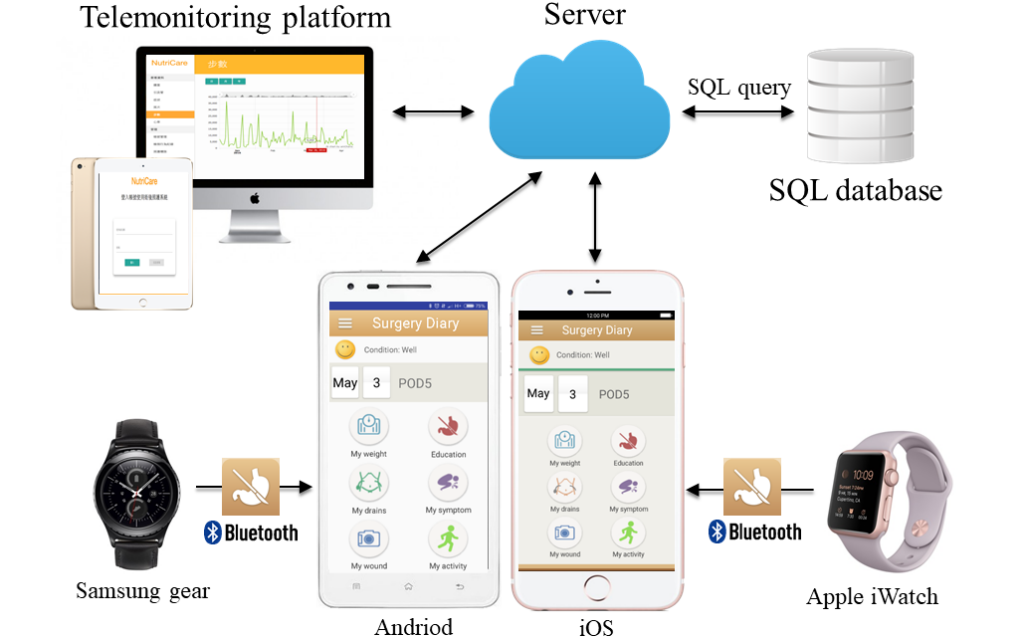
The system architecture: the wearable device for recording physical activity (number of steps) is Apple Watch for iOS and Samsung Gear S2 for Android. The wearable devices connected and sent data to the original customized apps in mobile phones, which were further connected to our designed app. SQL: standard database language.

SurgeryDiary is an iOS/Android app that facilitates patients who have undergone gastrectomy to access educational information of surgical procedures, record perioperative clinical variables (PA, associated discomfort, body weight, and drain amount), and transmit digital images of the surgical wound to the medical staff. PA (Figure 1, left) and heart rate (Figure 1, right) could be graphed for diverse study periods (ranging from days to years), and patients/caregivers could view the change in PA or heart rate by time increment. This app was developed by surgical professionals and software programmers to fulfill the need of the patients. The wearable device for recording PA (number of steps) is Apple Watch for iOS and Samsung Gear S2 for Android. Reportedly, both devices could reliably measure the number of steps as effective health evaluation indicators [39]. Figure 2 presents the system architecture. The wearable devices initially collected daily step count data, which were then transferred to the original customized wearable apps in the smartphones using the sync functionality. Furthermore, our designed app captured the data stored in the original customized wearable app. Next, data in our designed app were sent to the server when internet access was available for these smartphones. This function of interdevice data transmission worked well with no abnormal events reported by the patients. Furthermore, 1 case manager monitored the synchronous data during daytime on weekdays and would call the patients if their step counts had decreased or were completely missed. Patients continued to transmit data until postoperative day 28, and 1 medical staff who did not participate in designing this system independently reviewed and analyzed data.

### Outcomes

#### Feasibility: Protocol Completion

In this study, feasibility was defined as the proportion of participants using each element of the system (wearable device and uploading step counts) for at least 70% of the 28-day study period [40]. “Definitely feasible,” “possibly feasible,” and “not feasible” were defined as ≥70%, 50%-69%, and <50% participants meeting criteria, respectively.

#### Clinical Value: Association of Walking Steps and Early Discharge

The outcome of clinical value was to validate whether the improvement of PA based on wearable devices was a reliable parameter to estimate early discharge (length of hospital stay <9 days) in patients who had undergone gastrectomy. Our hypothesis was based on 1 report addressing that physical functional recovery, including adequate pain control, ability to mobilize, tolerance of oral intake, and no abnormal physical findings or laboratory tests, could be achieved on postoperative day 5 in patients with GC who had undergone gastrectomy; this was a parameter to predict if patients would qualify for early discharge [41]. Furthermore, we collected demographic (age, gender, body mass index, and comorbidities), type of gastrectomy, minimal invasive surgery, cancer histology, and cancer stage information for statistical adjustment.

## Results

### Participants’ Characteristics

Of the 43 analyzed patients (Table 1), 51% (22/43) were males, and the median age was 68 years, which is consistent with that observed in the population with GC in Taiwan. The median age of male and female patients was 72 years and 59 years, respectively. Among the study population, the median body weight was 65 kg and the median body mass index was 22.3. Regarding surgical procedures, there were 7 (16%) wedge resections, 30 (70%) subtotal gastrectomies, and 6 (14%) total gastrectomies. We observed a low value of serum albumin (<4.0 mg/dl; parameter of malnutrition) in 15 (35%) patients, and 22 (51%) patients had an ASA physical status classification system score of >2. Notably, 30 patients (70%) depended on the assistance of active caregivers to perform this task because of old age or limited prior experience with technology.

All questions received a mean rating over 4 out of 5, but 2 (5%) patients reported a rating of 3 for 1 question (“patient comfort using the wearable device”).

### Feasibility: Protocol Completion

All participants submitted step data every day in the first week. The weekly submission rate in the second, third, and fourth week was 93%, 91%, and 86%, respectively. The overall daily submission rate was 95.5% (1150 of days, given 43 subjects submitting data for 28 days). Of 54 missed transmission days, 6 occurred in the second week, 12 occurred in the third week, and 36 occurred in the fourth week. The leading reason for not sending data was that patients/caregivers forgot to charge the wearable devices (>90%).

### Clinical Value: Association of Walking Steps and Early Discharge

Table 2 outlines the comparison of study subjects with early discharge and without early discharge. There were 19 (44%) patients with early discharge. The patients with early discharge exhibited a significantly higher proportion of patients with minimal invasive surgery (68% vs 29%; *P*=.01), cancer histology of gastrointestinal stromal cancer (32% vs 4%; *P*=.01), and more walking steps on postoperative day 5 (5823 vs 4311; *P*=.01) compared with the patients without early discharge. Furthermore, we validated whether walking steps could predict early discharge using the logistic regression model (Table 3). We included significant variables on univariable analysis with a *P*<.05 into the multivariable analysis, which demonstrated that every 1000-step increment of walking on postoperative day 5 was associated with early discharge (odds ratio 2.72, 95% CI 1.17-6.32; *P*=.019) after adjustment of both baseline physical status and aforementioned variables.

**Table 1:** Demographic characteristics of study subjects, participant adherence to the protocol, and clinical outcomes.

Characteristic		Data (N=43)
Age, median (IQR^a^)		68.0 (58.0-75.0)
Male, n (%)		22 (51)
Body mass index, median (IQR)		22.3 (19.9-25.8)
Value of serum albumin (mg/dl) <4, n (%)		15 (35)
Smoking history, n (%)		39 (91)
American Society of Anesthesiologists score >2, n (%)		22 (51)
Minimal invasive surgery, n (%)		20 (47)
Charlson Comorbidity Index >2, n (%)		10 (23)
**Education level, n (%)**		
	Illiterate	1 (2)
	Up to high school	30 (70)
	>High school	12 (28)
**Employment level, n (%)**		
	Full-time employment	25 (59)
	Part-time employment	7 (16)
	Retired	10 (23)
	Unemployed	1 (2)
**Financial situation, n (%)**		
	Self-pay	41 (95)
	Social support	2 (5)
**Place of residence, n (%)**		
	Urban area	30 (70)
	Rural area	13 (30)
**Eastern Cooperative Oncology Group performance status scales, n (%)**		
	0	38 (88)
	1	5 (12)
**Method of gastrectomy, n (%)**		
	Wedge resection	7 (16)
	Subtotal gastrectomy	30 (70)
	Total gastrectomy	6 (14)
**Histological classification, n (%)**		
	Adenocarcinoma	36 (84)
	Malignant gastrointestinal stromal tumor	7 (16)
**System of American Joint Committee on Cancer staging, n (%)**		
	Stage I	15 (35)
	Stage II	20 (47)
	Stage III	8 (18)
	Major complication	6 (14)
**Method of participation, n (%)**		
	Caregiver	13 (30)
	Independent	30 (70)
**App** **system,** **n (%)**		
	Android	38 (88)
	iOS	5 (12)
**Participant compliance, n (%)**		
	At first week	43 (100)
	At second week	40 (93)
	At third week	39 (91)
	At fourth week	37 (86)
**Overall daily compliance**		
	Total submissions (person-day)	1204
	Days submitted, n (%)	1150 (95)
	Days missed, n (%)	54 (5)

^a^IQR: interquartile range.

**Table 2:** Comparison of the study subjects with early discharge and without early discharge.

Characteristic		Without early discharge (n=24)	With early discharge (n=19)	*P* value
Age, median (IQR^a^)		68.0 (58.5-74.5)	66.0 (56.0-77.0)	.84
Gender (male:female)		9:15	12:7	.09
Body mass index, median (IQR)		22.3 (20.9-23.6)	21.7 (19.8-28.1)	.90
Smoking history, n (%)		21 (88)	18 (95)	.42
Value of serum albumin (mg/dl) <4, n (%)		10 (42)	5 (26)	.29
American Society of Anesthesiologists score >2, n (%)		14 (58)	8 (42)	.29
Charlson Comorbidity Index >2, n (%)		4 (17)	6 (32)	.25
**Eastern Cooperative Oncology Group performance status scales, n (%)**				.67
	0	21 (88)	17 (90)	—^b^
	1	3 (12)	2 (10)	—
	Minimal invasive surgery, n (%)	7 (29)	13 (68)	.01
**Method of gastrectomy, n (%)**				.05
	Wedge resection	1 (4)	6 (32)	—
	Subtotal gastrectomy	19 (79)	11 (58)	—
	Total gastrectomy	4 (17)	2 (11)	—
**Cancer histology, n (%)**				.01
	Adenocarcinoma	23 (96)	13 (68)	—
	Gastrointestinal stromal cancer	1 (4)	6 (32)	—
**Method of participation, n (%)**				.40
	Caregiver	6 (25)	7 (37)	—
	Independent	18 (75)	12 (63)	—
	Walking steps (X1000) on postoperative day 5, median (IQR)	4.3 (4.1-4.7)	5.8 (4.5-6.1)	.01

^a^IQR: interquartile range.

^b^Not applicable.

**Table 3:** Adjusted multivariate analysis to predict early discharge.

Characteristic		Odds ratio (95% CI)	*P* value
Every 1000-step increment of walking		2.72 (1.17-6.32)	.02
Minimal invasive surgery		4.14 (0.71-24.03)	.11
**Method of gastrectomy (reference: wedge resection)**			
	Subtotal gastrectomy	0.40 (0.03-5.36)	.49
	Total gastrectomy	0.42 (0.01-13.26)	.63
**Cancer pathology (reference: adenocarcinoma)**			
	Gastrointestinal stromal cancer	1.03 (0.71-3.17)	.99
	Eastern Cooperative Oncology Group performance status scales (reference: 0)	0.58 (0.12-2.67)	.49

## Discussion

### Principal Findings

This study demonstrated the feasibility of using a mobile app connected to a wearable device to record perioperative numbers of steps in patients after major gastrectomy for GC. The PA variable based on the number of steps is a reliable parameter for predicting early discharge from the hospital. To the best of our knowledge, this is one of the innovative studies focusing on the development of comprehensive app functions to track the PA of cancer subjects [42], and we focus on the cancer patients undergoing gastrectomy. This system continues to monitor PA during the crucial perioperative period when several complications and functional/physical disorders occur and result in delayed recovery.

From our results, improved PA was associated with early discharge of the GC patients undergoing gastrectomy. It was because the patients with improved PA had resumed physical function, which was the main factor to evaluate if the patients were qualified to be discharged. One study investigating factors associated with early discharge of patients with GC undergoing gastrectomy found that several factors, such as improved PA, laboratory variables, minimally invasive surgery, and body temperature, could predict early discharge [43]. For the patients undergoing surgical procedures other than gastrectomy, the reports also supported the relationship between improved PA and early discharge [44,45]. Our study suggested that the surgical staff should regard PA as an important clinical parameter, which has previously been long ignored as research is often hindered by the challenge of adapting an easy, valid, and reliable measure to record PA [6]. However, because of the relatively small sample, the results of the statistical tests should be interpreted with care; more patients should be enrolled to validate the relationship between improved PA and early discharge.

Although medical professionals acknowledge the significance of PA in the prevention of GC and maintenance of chronic diseases [3,46], few incorporate PA counseling into routine clinic visits/care [47]. The gap between knowledge and implementation of PA in daily practice can be attributed to several reasons, including transitions of care from an inpatient stay to the community, labor of self-recording, and difficult access for medical professionals to check data. The ability to wirelessly interface with wearable and mobile devices and the application of platforms/apps provide surgical patients with a method to share health information with their surgeons. Our app is unique in the breadth and convenience of PA data that it can capture.

In contrast to the rapid growth of the field of medical mHealth, research on surgical subjects who are in much need of continuous care because of a marked decline in general conditions is limited. Among surgical mHealth, the application of wound care is the main topic for validating its clinical effect [48-50] because wound care is a unique issue for surgical patients in comparison with medically ill patients, and telephone conversations or questionnaires cannot be used to access the visual component. In addition to our developed system, a recent study used another wearable device to track step counts of patients who had undergone diverse abdominal surgeries within 1 month after discharge and showed that the mHealth app could effectively track recovery [27]. Both studies established the feasibility of PA generated by wearable devices, which should be routinely implemented in clinical services and daily life to monitor the degree of recovery of surgical patients. However, our system monitored the step counts specifically in patients who had undergone gastrectomy immediately after gastrectomy, which provided more detailed perioperative data of PA for further analysis. In this study, we developed an app that is compatible with both Android and iOS devices to enable its use with any type of mobile devices. Another strength of our study is the largest cases series addressing the use of mHealth app in the GC patients to date.

Several studies have used electronic assessment of patient-report outcomes, which has proved to be as accurate as paper and pencil administration in 1 multicenter observational cohort study [51,52]. By collecting information that was unavailable and data that were unavailable in the traditional process of perioperative care, both health care organizations and medical staff can undertake quality improvement initiatives and conduct a comprehensive analysis. Conversely, health care workers in Taiwan are exposed to high levels of occupational stress and heavy workloads [53]. Although PA is a reliable predictor of outcomes for surgical patients, collection of data and records can be another burden for health care workers. At our hospital, we have considered implementing our electronic PA system with the health information system in the future, to attenuate workloads of the medical staff and expedite data extraction and analysis.

### Limitations

This study should be evaluated in the context of some limitations. First, this study was conducted in a single medical center. The findings of this study might be specific to our subjects, but they warrant additional research in other populations. Thus, we will design a prospective randomized clinical trial to validate the efficacy of this app in patients with GC who are undergoing gastrectomy (a study group treated by wearable devices connected to the research program and a control group treated by wearable devices who are not affiliated with the research program). Second, 4.5% of patient days exhibited a technological problem that hindered data collection mostly because of the issue of forgetting to charge the battery of the wearable devices. Usually, patients needed to charge the battery every 2 to 3 days, depending on which wearable device they used; in future, devices should be improved, particularly for their battery capacity. Third, a connection between this app and other types of wearable devices was the main barrier that limited the number of participants. Currently, we provided a wearable device to patients, but continuous modification of the system is essential with upgrades of the software and associated devices, including smartphones and wearable devices. Finally, in our study, the metric for PA is daily step count. Moderate to vigorous PA (MVPA) is also an important parameter for assessing PA in surgical patients in conjunction with daily step counts. However, MVPA is relatively more stressful for early postoperative patients compared with simple measures of daily step counts. In future studies, we will validate the role of MVPA on late postoperative surgical patients as they recover to competent physical and mental functions.

### Conclusions

The use of mobile apps implementing wearable devices for recording PA in patients with postoperative GC was feasible in patients undergoing gastrectomy in this study. With the support of the mHealth platform, this app offers seamless tracing of patients’ recovery with a little extra burden and turns subjective PA into an objective, measurable parameter.

## Abbreviations

ASA: American Society of Anesthesiologists
GC: gastric cancer
ICD-9-CM: International Classification of Diseases, 9th Revision, Clinical Modification
ICU: intensive care unit
mHealth: mobile health
MVPA: moderate to vigorous physical activity
PA: physical activity
